# Atraumatic Splenic Rupture in *Legionella pneumophila* Pneumonia

**DOI:** 10.1155/2023/9625170

**Published:** 2023-06-07

**Authors:** Elliott Worku, Dominic Adam Worku

**Affiliations:** ^1^Adult Intensive Care Services, The Prince Charles Hospital, Chermside, Queensland, Australia; ^2^Infectious Diseases Department, Morriston Hospital, Swansea, UK; ^3^Public Health Wales, Cardiff, UK

## Abstract

A previously fit 46-year-old male handyman presented to a rural hospital with a cough, fever, and epigastric pain without peritonism. The patient was admitted medically with symptoms and radiological appearances consistent with atypical community-acquired pneumonia. During the first 48 hours of admission, he suffered a significant haemodynamic deterioration and was transferred to the intensive care unit (ICU) for vasoactive support. Following stabilisation, urgent abdominal CT imaging demonstrated splenic rupture with haematoma in the absence of historical trauma. Emergency splenectomy was performed; the histopathological examination was unremarkable. Investigations for the presenting complaint confirmed *Legionella pneumophila* serotype 1 pneumonia by urinary antigen testing. The patient was extubated on postoperative day 2 and stepped down from ICU to complete a 14-day course of azithromycin. Atraumatic splenic rupture is a rarely described clinical entity. The process can be subdivided into pathological and nonpathological (spontaneous) cases. Pathological atraumatic splenic rupture may occur in the context of wide-ranging aetiologies, including bacterial pneumonia; however, the association with *Legionella pneumophila* serotype 1 is exceptional, with this representing the eighth case in the medical literature.

## 1. Introduction

Community-acquired pneumonia (CAP) is a leading cause of hospital admissions, mortality, and health-related cost worldwide [1]. While *Streptococcus pneumoniae* is the leading cause of pneumonia worldwide, approximately 15–20% of CAPs are secondary to atypical organisms [2]. Atypical pneumonias are named such due to their relative resistance to beta-lactam therapy (resulting from a lack of a bacterial cell wall), their intracellular life cycle, their inability to be cultured or gram stained using traditional methods, their diverse symptomatology, and their propensity for extrapulmonary disease. Of these atypical pathogens, *Legionella* pneumonia is the most common [3, 4].

Clinically, *Legionella* infection may present with Pontiac fever or pneumonia. Pontiac fever is an acute self-limiting febrile illness with a paucity of respiratory tract signs; [5–7] *Legionella* pneumonia however presents 2–10 days postexposure with dry cough on the background of a prodrome of headache, myalgia, arthralgia, diarrhoea, and relative bradycardia. Chest X-ray findings may range from isolated pulmonary infiltrates to well-circumscribed lesions and cavitation [8, 9]. Clinical differentiation from other causative pathogens of CAP may be suggested by the presence of haemoptysis, transaminitis, or hyponatraemia due to syndrome of inappropriate ADH release (SIADH) [8].

One important but infrequently described extrapulmonary association of Legionnaire's pneumonia is atraumatic splenic rupture (ASR). ASR is a rarely described condition which may be classified into pathological (93%) and idiopathic (7%) aetiologies [10]. In pathological cases where a cause is identified, the majority may be subscribed to three different disease processes: namely, hematologic malignancy (16.4%) e.g., acute leukaemia; infection (14.8%) e.g., Epstein–Barr virus (EBV); and inflammatory (10.9%) causes such as chronic pancreatitis and amyloidosis [10].

Idiopathic ASR is defined according to the Orloff and Peskin criteria [11]. These comprise an absence of trauma and perisplenic adhesions and normal macroscopic/histological examinations of the abdominal viscera including the spleen. More recently, a fifth criterion of negative serology for relevant viral infections has been proposed [12].

Management of ASR can vary from conservative management, to interventional radiological techniques such as arterial embolization, and in more catastrophic cases, emergent laparotomy and total splenectomy. The decision regarding embolization Vs surgical approaches may be guided by logistical factors, such as local interventional radiology availability, transport times to referral centres, and the degree of patient haemodynamic instability [12]. In this case report, we present a case of ASR in a 46-year-old noncomorbid gentleman diagnosed with *Legionella pneumophila* pneumonia. This is the eighth recorded case in the literature.

## 2. Case Report

A 46-year-old Caucasian male with no past medical history, regular medications, or illicit drug use presented to a rural emergency department with five days history of rigors, night sweats, and unrelenting cough despite a course of oral antibiotics provided by their primary care physician.

The patient reported a 15-pack year smoking history and avian contact in the weeks preceding his illness. The patient was a handyman by occupation with duties including water system maintenance, but had no recent foreign travel.

**Figure 1:**
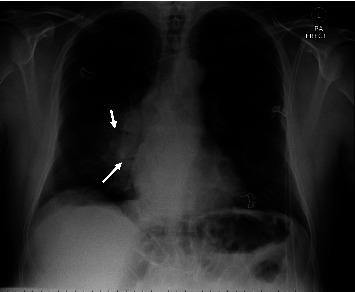
CXR on admission demonstrating dense right perihilar consolidation with associated air bronchograms (white arrows).

On presentation, the patient was febrile (40.1°C), diaphoretic, and tachycardic (sinus rhythm, 140 beats per minute), but normotensive (140/70 mmHg). He was tachypnoeic (20–30 bpm), saturating 94% on room air, with a productive cough and streaks of haemoptysis. Plain chest radiograph performed in the emergency department revealed right perihilar consolidation and air bronchograms, consistent with clinically auscultated crepitations over the right lower lung field. The diagnosis of community-acquired pneumonia (CAP) was made (Figure 1). The patient reported some mild abdominal discomfort, with palpation positive for LUQ and epigastric tenderness, without peritonism, guarding, nor organomegaly. There were no stigmata of infective endocarditis nor exanthem. His SMART-COP score was 2 on admission with a CURB65 score of 1.

After crystalloid fluid resuscitation, benzylpenicillin and doxycycline were administered per local guidance, providing coverage for typical and atypical causes of CAP. The patient was referred and admitted under the general medical team for ongoing treatment. Respiratory virus testing, sputum culture, and blood cultures were requested along with urinary pneumococcal and *Legionella* urinary antigens. Serological testing for atypical pathogens including *Mycoplasma pneumoniae* and *Chlamydia psittaci* was also sought in view of avian exposure, and the presence of extrapulmonary symptoms. Infectious mononucleosis was ruled out with nonreactive viral capsid antigen IgM and negative monospot with cytomegalovirus and HIV serology yielding negative results.

**Figure 2:**
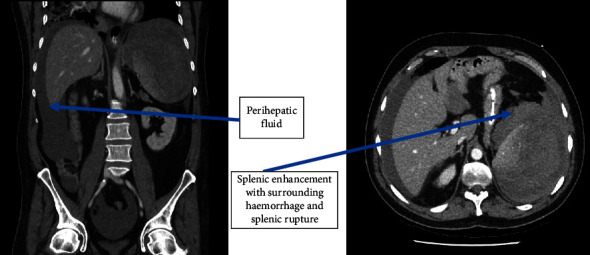
CT-abdomen and pelvis on day 3 of admission demonstrating large splenic laceration, haematoma, and haemoperitoneum.

24 hours later, the patient was attended by the medical emergency response team for a new episode of hypotension (BP 86/51 mmHg) and worsening tachypnoea during which high-flow nasal oxygen was required. Due to progressive deterioration, an intensive care unit (ICU) opinion was sought, and he was transferred to the intensive care unit (ICU) for haemodynamic support presumed secondary to severe respiratory sepsis. Repeat haemoglobin measurement on admission to the ICU was decreased now to 94 g/L (haematocrit of 0.27) from 133 g/L one day prior. In the absence of melaena, injury, peritonism, or suggestion of haemolysis, this decrease was initially attributed to haemodilution. Lactate per arterial blood gas (ABG) was slightly increased at 2.1 mmol/L. Central venous access was obtained and vasopressors commenced; however, there was a rapid escalation in noradrenaline and subsequent vasopressin infusions, and thus stress dose hydrocortisone was supplemented for the presumption of severe vasoplegic septic shock. Due to this deterioration, antimicrobial therapy was escalated to piperacillin/tazobactam 4.5 g TDS and IV azithromycin 500 mg OD as per local guidelines for severe CAP. Following initial stabilisation, four hours into intensive care admission the patient decompensated suddenly with a frankly peritonitic abdomen. They required endotracheal intubation for humanitarian reasons and worsening type 1 respiratory failure. Urgent computed tomography pulmonary angiogram (CTPA) and contrast abdominal-pelvis CT were performed posthaemodynamic resuscitation, demonstrating multilobar pulmonary consolidation, with large volume perihepatic fluid, and heterogeneous splenic enhancement consistent with laceration and haemoperitoneum (Figure 2). In the absence of a trauma history, abrupt and progressive haemodynamic instability and radiological findings a diagnosis of ASR was made.

Arterial blood gas analysis performed postreturn from the imaging department demonstrated severe raised anion gap metabolic acidosis and anaemia: pH 7.03, lactate 13.5 mmol/L, BE −19.5 mEq/L, and Hb 63 g/L. The massive transfusion protocol was activated, while arrangements were made for emergent transport to the operating theatre. At laparotomy, intraoperative findings confirmed capsular rupture of the spleen, and 4 L of altered blood product was retrieved from the abdomen, following which the surgical team proceeded to perform a total splenectomy. Macroscopic and histopathological examination revealed a spleen of normal architecture, weighing 137 g, with no evidence of malignant infiltration.

Postoperatively, the metabolic acidosis rapidly improved, with a minimal transfusion requirement of 2 further packed red cell units in the following 24 hours. On the second postoperative day, the patient was successfully extubated and liberated from all haemodynamic supports. On day 3, urinary antigen for *Legionella pneumophila* returned positive, and thus, azithromycin was continued to complete a 14-day course. The patient recovered uneventfully postsplenectomy and received the requisite vaccinations and ongoing antimicrobial prophylaxis as per national guidance and was discharged home.

## 3. Investigations

Initial lab tests (Table 1) revealed leucocytosis with neutrophil predominance, mild lymphopenia, and no peripheral eosinophilia. Inflammatory markers were elevated, but venous lactate was normal at 1.5 mmol/L. The patient had serum sodium at the lower limit of normal, mild acute kidney injury, and transaminitis. A rapid severe acute respiratory syndrome coronavirus 2 (SARS-CoV-2) antigen test was negative. In the setting of his occupation, the possibility of Legionnaire' pneumonia was raised.

## 4. Discussion

On admission, our patient was determined to have low CURB65 and SMART-COP scores which are prognostic in the setting of typical CAP. The CURB65 score is a validated scoring system that helps determine the appropriate disposition of patients (inpatient treatment vs. outpatient) and mortality risk. The score of 1 recorded in this patient was supportive of a low-risk course, appropriate for outpatient management [13]. The SMART-COP score is a more sensitive scoring system in predicting the need for intensive care management, despite which our patient was designated again as low risk. Importantly, neither scoring systems are validated in atypical CAP [13, 14] nor inform of the risks of rare complications such as atraumatic splenic rupture. Some 20–80% of patients with *Legionella* pneumonia progress to requiring intensive care support, and clinical deterioration can occur shortly after admission, as was the case in our patient. Mortality with timely investigation and management is reportedly 5–30%, but may be as high as 80% in immunosuppressed individuals experiencing treatment delays [15, 16].

Splenic rupture is a surgical emergency and may present with abdominal pain with radiation to the left shoulder tip (Kehr's sign) [17]. This can be quickly accompanied by haemodynamic instability due to life-threatening intra-abdominal haemorrhage. Unfortunately, clinical signs may be difficult to elicit in sedated, intubated patients in intensive care. In a series of 8 cases of atraumatic splenic ruptures of varying aetiologies, all patients had splenomegaly present (>200 g in weight or 11 × 7 × 5 cm in size) with common signs and symptoms of nausea, bowel obstruction, delirium, and peritonism (*n* = 4); however, Kehr's sign was rarely present (*n* = 2) [18]. In the setting of hypotension of unclear cause, with an associated reduction in haemoglobin, surgical causes including ASR should be considered [19]. This may also present atypically with thoracic back pain and right upper quadrant pain presumably due to haemoperitoneum [20]. While there was no prior abdominal imaging in our patient to exclude preexisting splenic pathology, the normal histopathology at the operation makes this unlikely. Our patient exhibited mild left upper quadrant and epigastric pain on presentation, but without attendant peritonism or guarding, and this was not considered sufficient to prompt targeted radiological investigation in the emergency department or during medical admission. The later development of Kehr's sign following admission to intensive care was coincident with substantial haemodynamic deterioration. This clinical emergency required resuscitation and stabilisation measures prior to performance of CT imaging being practicable or safe. While there was potentially a diagnostic delay, there is a lack of widespread appreciation of this rare complication of atypical pneumonia, and in the absence of trauma or convincing abdominal signs there was limited clinical suspicion for splenic rupture. Furthermore, at presentation, there may have simply been pain related to splenic capsular stretch from splenomegaly rather than rupture. It is possible that abdominal imaging on presentation may not have yielded findings which would have altered the management course and that rupture may have only been provoked by coughing in the setting of pneumonia or Valsalva efforts later in the admission. Indeed, in patients with ASR, approximately a third of cases present with shock, yet the diagnosis is often made postmortem [21]. It would seem prudent as awareness of this entity grows, in the presence of left upper quadrant abdominal pain and pneumonia, that abdominal ultrasound be performed to facilitate early identification of patients at risk of ASR [22].

In all seven cases of ASR complicating *Legionella* pneumonia identified in a literature search, *Legionella pneumophila* serotype 1 was the causative species (Table 2) [23–29]. While this may be due to specific virulence factors, it may simply reflect testing bias due to the global adoption of the urinary *Legionella* antigen test as the main method for diagnosing *Legionella* infection. The urinary antigen test is an ELISA, with reported sensitivity of 94.6% and specificity of 100% for diagnosing *Legionella pneumophila* serotype 1 but is of limited utility with respect to other pathogenic *Legionella* species such as serotype 2 and *longbeachae* [30, 31]. Of the 7 previous cases we identified (Table 2), 4 used the urinary antigen test with the remainder utilising direct immunofluorescence which is both technically challenging and of limited sensitivity [32]. In one instance, polymerase chain reaction (PCR) was also used to make the diagnosis. This is a highly specialised investigation and is not routinely available in most centres; however, PCR can be used on a wide range of clinical samples (e.g., blood, bronchoalveolar lavage fluid, and sputum) and can detect all relevant *Legionella* species and serogroups and thus may become a gold standard test in the future [33]. Interestingly, in all previously identified cases of ASR complicating *Legionella* infection, patients were male with an average age of 55 years (range 42–70 years). The majority (71%) had no predisposing comorbidities for *Legionella* infection, in keeping with our report. The remaining two cases were immunocompromised by diabetes mellitus (*n* = 1) and methotrexate use (*n* = 1), of whom one case died [26, 29].

The interval between the onset of illness and ASR varied but was often within the first week of illness (median 6 days). However, in one instance, splenic rupture occurred 3 weeks after apparently successful treatment for *Legionella* disease, highlighting the possible need to counsel patients regarding this complication as we do patients with infectious mononucleosis [29]. Overall survival of these identified cases was 75% (*n* = 6/8) with all cases being managed surgically and receiving appropriate antibiotic regimes. In those who survived, there was a paucity of follow-up data, limiting inferences about long-term outcomes. In the two cases that died, diagnostic delay, uncontrolled bleeding, and severe comorbidities including immunocompromise and cardiomyopathy were likely contributory to poor outcomes [23, 26]. In one instance, coinfection with H1N1 influenza was noted alongside vascular thrombosis which may have precipitated splenic injury by means of portal hypertension [28].

While it remains unclear which risk factors are most relevant to ASR pathogenesis in the setting of *Legionella* pneumonia, in a guinea pig model there was a clear relationship between *Legionella* inoculum size, disease severity, and the occurrence of death [34]. Potentially, the same holds true for the immunologic insult leading to ASR. ASR has been described in multiple settings in which malignancy, viral infection, inflammatory and iatrogenic causes (e.g., antiplatelets and anticoagulants) predominate [11, 35]. Other at risk groups include women during late pregnancy (3^rd^ trimester) or those with ectopic pregnancies in whom this differential should be considered given the raised intra-abdominal pressure from the gravid uterus [36]. More recently, ASR has been described in both children and adults with SARS-CoV-2 of severity ranging from asymptomatic to requiring invasive mechanical ventilation [37, 38]. The pathophysiology is thought to be from COVID-19-induced lymphocyte necrosis, follicle depletion, splenic blood vessel congestion, and vascular proliferation with resultant haemorrhage [39]. As such, SARS-CoV-2 testing should be included as part of the viral serological testing in patients presenting with ASR.

Of the four cases of *Legionella*-associated ASR where histopathological examination of the spleen was performed, findings ranged from scanty polymonorphonuclear infiltrate [23], splenomegaly [25], congested inflamed red pulp (splenitis) [27], and diffuse splenic coagulation [28]. While in our patient histological analysis was determined normal, it is unclear if interobserver variability or insufficient sampling may explain these findings. The underlying mechanism of ASR in *Legionella* likely involves downstream aberrant immunological responses with splenic hyperplasia, coagulopathy from septicaemia, and venous congestion from associated cold agglutinin formation. In this setting, any sudden increase in portal pressure from coughing or abdominal palpation may precipitate rupture [40].

Given the characteristics of *Legionella*, appropriate antimicrobial agents must be able to penetrate the lung parenchyma and achieve high intracellular concentrations. Antibiotic classes which have inherent activity against *Legionella* include the macrolides, rifamycins, tetracyclines, and quinolones [8]. Levofloxacin and Azithromycin are first-line therapies favoured by the BTS (British Thoracic Society) and IDSA (Infectious Diseases Society of America), respectively, given their excellent pharmacokinetics and inherent bactericidal activity. When comparing quinolone and macrolide efficacy in *Legionella* infection, a recent highly powered systematic review found there to be no significant difference in mortality (6.88% versus 7.43% and *p*=0.66) or overall treatment success between the two groups [41]. Indeed, in most clinical, outcomes there was no significant difference between the two classes of antibiotics although fluoroquinolones were associated with reduced complication rates including respiratory failure and subsequent Clostridioides difficile infection which is a major consideration given its associated morbidity and mortality [41]. While resistance to these agents in *Legionella* infection has rarely been reported, this may become an issue in the future given the empiric use of *Legionella* active therapy in the initial management of CAP globally and is worthy of future attention. While the recommended duration of therapy varies, it should be typically provided for 5–10 days but this may be extended up to 21 days in those who are immunosuppressed or with severe disease, as evidenced in our case by the need for both intensive care admission and splenectomy [32].

## 5. Conclusion

This report adds to the small number of cases of *L. pneumophila* 1 pneumonia complicated by ASR and highlights the importance of Hickams' Dictum and multidisciplinary consultations. In patients with *Legionella* pneumonia and left upper quadrant pain, ASR should be considered and investigated. Using traditional criteria for operative intervention, patients with ASR are more likely to require splenectomy. With timely diagnosis and prompt surgical intervention, a favourable outcome can be achieved.

## Figures and Tables

**Table 1:** Admission blood tests and SMART-COP score.

Parameter	Result
White cell count (WCC)	13.5 × 10^9^/L (3.6–11 × 10^9^/L)
	89.8% neutrophils, 0.6 × 10^9^/L lymphocyte count
C-reactive protein (CRP)	396 mg/l (<5 mg/L)
Haemoglobin	152 g/L (130–180 g/L)
Haematocrit	0.43 (0.4–0.54 I/I)
Creatinine	114 *μ*mol/L (59–104 *μ*mol/L)
Urea	5.7 mmol/L (2.5–7.8 mmol/L)
Sodium	133 mEq/L (133–146 mEq/l)
Phosphate	0.46 mEq/L (0.8–1.50 mEq/l)
Gamma-glutamyl transferase	183 U/L (<61 U/L)
Alanine transaminase	50 U/L (<50 U/L)
Aspartate aminotransferase	71 U/L (<50 U/L)
Alkaline phosphatase	136 U/L (30–130 U/L)
Bilirubin	20 *μ*mol/(<21 *μ*mol/L)
SMART-COP score	2
Human immunodeficiency virus (HIV) serology	Negative
Cytomegalovirus IgG/IgM	Negative
Monospot test	Negative
Blood film	Leucocytosis
*Chlamydia psittaci* serology	Negative
*Mycoplasma* serology	Negative

**Table 2:** Identified cases of atraumatic splenic rupture (ASR) in association with confirmed *Legionella* pneumonia (*n* = 7) in [23–29].

Age (gender)	Year	Time from symptom onset to rupture	Radiological features	Treatment	Legionella score	Outcome
(1) 63 (male	1990	11 days	Left sided consolidation	Erythromycin + benzylpenicillin	3	Died [23]
(2) 42 (male)	1993	4 days	Left sided consolidation	Penicillin	0	Survived [24]
(3) 52 (male)	1996	8 days	Left lower lobe consolidation	Cefotaxime + clarithromycin	2	Survived [25]
(4) 70 (male)	2004	6 days	Bilateral chest infiltrates	Cefotaxime + erythromycin Clarithromycin + levofloxacin	1	Died [26]
(5) 47 (male)	2010	1 day	Right upper lobe consolidation	Rifampicin + clarithromycin	3	Survived [27]
(6) 42 (male)	2012	—	Bilateral lung infiltrates right	Ceftriaxone + levofloxacin	1	Survived [28]
(7) 69 (male)	2021	24 days	Lower lobe pneumonia	Clarithromycin + levofloxacin	0	Survived [29]

## References

[1] Regunath H., Oba Y. (2022). *Community-Acquired Pneumonia*.

[2] Lynch J. P., Zhanel G. G. (2009). Streptococcus pneumoniae: epidemiology, risk factors, and strategies for prevention. *Seminars in Respiratory and Critical Care Medicine*.

[3] Cunha B. A. (2006). The atypical pneumonias: clinical diagnosis and importance. *Clinical Microbiology and Infection*.

[4] Miyashita N. (2022). Atypical pneumonia: pathophysiology, diagnosis and treatment. *Respiratory Investigation*.

[5] Yu V. L., Ramirez J., Roig J., Sabria M. (2004). Legionnaires disease and the updated IDSA guidelines for community‐acquired pneumonia. *Clinical Infectious Diseases*.

[6] Ambrose J., Hampton L. M., Fleming-Dutra K. E. (2014). Large outbreak of legionnaires’ disease and Pontiac fever at a military base. *Epidemiology and Infection*.

[7] Edelstein P. H., Roy C. R. (2015). Legionnaires’ disease and pontiac fever. *Mandell, Douglas, and Bennett’s Principles and Practice of Infectious Diseases*.

[8] Cunha B. A. (2010). Legionnaires’ disease: clinical differentiation from typical and other atypical pneumonias. *Infectious Disease Clinics of North America*.

[9] Muder R. R., Yu V. L., Parry M. F. (1987). The radiologic manifestations of legionella pneumonia. *Seminars in Respiratory Infections*.

[10] Renzulli P., Hostettler A., Schoepfer A. M., Gloor B., Candinas D. (2009). Systematic review of atraumatic splenic rupture. *British Journal of Surgery*.

[11] Labaki M. L., De Kock M. (2022). Atraumatic splenic rupture in a patient treated with Rivaroxaban: a case report and a narrative review. *Clinical Case Reports*.

[12] Crate I. D., Payne M. J. (1991). Is the diagnosis of spontaneous rupture of a normal spleen valid?. *Journal of the Royal Army Medical Corps*.

[13] Lim W. S., Baudouin S. V., George R. C. (2009). British Thoracic Society guidelines for the management of community acquired pneumonia in adults: update 2009. *Thorax*.

[14] Charles P. G. P., Wolfe R., Whitby M. (2008). SMART-COP: a tool for predicting the need for intensive respiratory or vasopressor support in community acquired pneumonia. *Clinical Infectious Diseases*.

[15] Huh J. Y., Choi S. H., Jo K. W. (2022). Incidence and risk factors associated with progression to severe pneumonia among adults with non-severe legionella pneumonia. *Acute and Critical Care*.

[16] Kao A. S., Myer S., Wickrama M., Ismail R., Hettiarachchi M. (2021). Multidisciplinary management of Legionella disease in immunocompromised patients. *Cureus*.

[17] Akoury T., Whetstone D. R. (2022). *Splenic Rupture*.

[18] Liu J., Feng Y., Li A., Liu C., Li F. (2019). Diagnosis and treatment of atraumatic splenic rupture: experience of 8 cases. *Gastroenterology Research and Practice*.

[19] White D. Atraumatic Splenic Rupture. *Critical Care*.

[20] Sforza C., Margelli M., Mourad F., Brindisino F., Heick J. D., Maselli F. (2023). Spontaneous spleen rupture mimicking non-specific thoracic pain: a rare case in physiotherapy practice. *Physiotherapy Theory and Practice*.

[21] Ahbala T., Rabbani K., Louzi A., Finech B. (2020). Spontaneous splenic rupture: case report and review of literature. *The Pan African medical journal*.

[22] Andrews M. W. (2000). Ultrasound of the spleen. *World Journal of Surgery*.

[23] Holmes A., Ng V., Fogarty P. (1990). Spontaneous rupture of the spleen in Legionnaires’ disease. *Postgraduate Medical Journal*.

[24] Saura P., Valles J., Jubert P., Ormaza J., Segura F. (1993). Spontaneous rupture of the spleen in a patient with legionellosis. *Clinical Infectious Diseases*.

[25] Domingo P., Rodríguez P., López-Contreras J., Rebasa P., Mota S., Matias-Guiu X. (1996). Spontaneous rupture of the spleen associated with pneumonia. *European Journal of Clinical Microbiology & Infectious Diseases*.

[26] Trisolini R., Agli L. L., Cancellieri A. (2004). Bronchoalveolar lavage findings in severe community-acquired pneumonia due to Legionella pneumophila. *Respiratory Medicine*.

[27] Casanova-Roman M., Casas J., Sanchez-Porto A., Nacle B. (2010). Spontaneous rupture of the spleen associated with Legionella pneumonia. *The Canadian Journal of Infectious Diseases & Medical Microbiology*.

[28] Mastroianni C., Citton R., Del Borgo C., Belvisi V. (2012). Pandemic influenza H1N1, legionellosis, splenic rupture and vascular thrombosis: a dangerous cocktail. *Journal of Postgraduate Medicine*.

[29] Yadav N., Sundararajan K. (2021). Its never too late-spontaneous rupture of spleen and life threatening hypovolemic shock in a patient recuperating from legionnaire’s disease. *Journal of Anaesthesia & Crit Care Case Reports*.

[30] Lebrun L., Tram C., Lapierre F., Grangeot-Keros L., Pillot J. (1983). Detection of *Legionella pneumophila* antigenby elisa in urine of experimentally infected Guinea-pigs. *Annales de l’Institut Pasteur/Microbiologica*.

[31] Garbino J., Bornand J. E., Uckay I., Fonseca S., Sax H. (2004). Impact of positive legionella urinary antigen test on patient management and improvement of antibiotic use. *Journal of Clinical Pathology*.

[32] Sharma L., Losier A., Tolbert T., Dela Cruz C. S., Marion C. R. (2017). Atypical pneumonia: updates on Legionella, chlamydophila, and Mycoplasma pneumonia. *Clinics in Chest Medicine*.

[33] Avni T., Bieber A., Green H., Steinmetz T., Leibovici L., Paul M. (2016). Diagnostic accuracy of PCR alone and compared to urinary antigen testing for detection of legionella spp.: a systematic review. *Journal of Clinical Microbiology*.

[34] Twisk-Meijssen M. J., Meenhorst P. L., van Cronenburg B. J., Mulder J. D., Scheffer E., van Furth R. (1987). The course of legionella pneumonia in Guinea pigs after inhalation of various quantities of L.pneumophila. *Immunobiology*.

[35] Arshad M. F., Javed N., Karim S. M., Ahmad E., Abid N. U. A. (2018). Atraumatic splenic rupture after myocardial infarction. *European Journal of Case Reports in Internal Medicine*.

[36] Li W., Yin D., Huo N. C., Wang X. Y., Zhang S. L. (2013). A case of spontaneous splenic rupture in an ectopic pregnancy. *Journal of Obstetrics and Gynaecology*.

[37] Bakalli I., Biqiku M., Cela D. (2022). Atraumatic splenic rupture in a child with COVID 19. *BMC Pediatrics*.

[38] Shaukat I., Khan R., Diwakar L., Kemp T., Bodasing N. (2021). Atraumatic splenic rupture due to covid-19 infection. *Clinical Infection in Practice*.

[39] Feng Z., Diao B., Wang R. (2020). The Novel Severe Acute Respiratory Syndrome Coronavirus 2 (SARS-CoV-2) Directly Decimates Human Spleens and Lymph Nodes. https://www.medrxiv.org/content/10.1101/2020.03.27.20045427v1.

[40] Guy S., De Clercq S. (2016). Splenic rupture in community acquired pneumonia: a case report. *International Journal of Surgery case reports*.

[41] Jasper A. S., Musuuza J. S., Tischendorf J. S. (2021). Are fluoroquinolones or macrolides better for treating legionella pneumonia? A systematic review and meta-analysis. *Clinical Infectious Diseases*.

